# Masked Morphological Priming and Sensitivity to the Statistical Structure of Form–to–Meaning Mapping in L2

**DOI:** 10.5334/joc.221

**Published:** 2022-05-09

**Authors:** Eva Viviani, Davide Crepaldi

**Affiliations:** 1 International School for Advanced Studies (SISSA), Via Bonomea 265, 34136, Trieste, Italy; 2 University of Oxford, Oxford, England

**Keywords:** Bilingualism, Morphology, Masked priming, Language proficiency

## Abstract

In one’s native language, visual word identification is based on early morphological analysis and is sensitive to the statistical structure of the mapping between form and meaning (Orthography–to–Semantic Consistency, OSC). How these mechanisms apply to a second language is much less clear. We recruited L1 Italian–L2 English speakers for a masked priming task where the relationship between prime and target was morphologically transparent, e.g., *employer–EMPLOY*, morphologically opaque, e.g., *corner-CORN*, or merely orthographic, e.g., *brothel–BROTH*. Critically, participants underwent thorough testing of their lexical, morphological, phonological, spelling, and semantic proficiency in their second language. By exploring a wide spectrum of L2 proficiency, we showed that this factor critically qualifies L2 priming. Genuine morphological facilitation only arises as proficiency grows, while orthographic priming shrinks as L2 competence increases. OSC was also found to modulate priming and interact with proficiency, providing an alternative way of describing the transparency continuum in derivational morphology. Overall, these data illustrate the trajectory towards a fully consolidated L2 lexicon and show that masked priming and sensitivity to OSC are key trackers of this process.

## 1. Introduction

Visual word identification occurs effortlessly in skilled adult readers. This process has received a considerable amount of attention, and there is now wide consensus that the recognition of printed words involves an early morphological analysis – words that are made up of meaningful sub–parts, such as *kind-ness* or *clean-er*, are identified via their constituents (e.g., Amenta and Crepaldi, 2012). Masked priming experiments have further revealed that morpheme identification is primarily based on form, as indicated by the fact that even pseudoderived words, like *corner*, facilitate the identification of their pseudostems, *corn*, more than orthographic controls (e.g., *dialog-DIAL* Grainger et al., 1991; Kazanina, 2011; Longtin et al., 2003; Lavric et al., 2007; Marelli et al., 2013; Rastle et al., 2000, 2004) (but see Milin et al., 2017, for conflicting evidence). Some models of word identification interpret these effects as related to an early stage in morphological decomposition that is semantically blind (e.g., Crepaldi et al., 2010; Rastle et al., 2004; Taft and Nguyen-Hoan, 2010), although some masked priming studies reported that facilitation is greater in transparent than opaque^1^ pairs (e.g., Diependaele et al., 2011; Feldman et al., 2009, 2012; Marelli et al., 2013), suggesting the involvement of some early semantic processing that would stem from morpho-orthographic segmentation.

Whether the same mechanisms apply to visual word identification in a second language (L2) is far less clear. The few experiments which have investigated this diverge in both data and theoretical interpretations. Silva and Clahsen (2008) investigated masked morphological priming with derived words (e.g., *bitterness*) in a group of L1–English, and in different groups of advanced L2–English readers. They compared derivational with repetition priming (*rigidity–RIGID* vs. *rigid–RIGID*), and found that the two effects are equally strong in L1, but not in L2. Based on these results, the authors argue that L2 readers might only have partial access to the combinatorial processes that are necessary to appreciate morphology as in their L1, and they would therefore rely more on whole-word retrieval in visual word recognition.

Working with prefixed, (pseudo)derived primes (e.g., *disagree–AGREE, mischief–CHIEF, stranger–ANGER*) and with Chinese-English bilinguals, Li and Taft (2019) similarly found a difference between morphological priming in L1 and L2. Although L1 readers experienced facilitation with transparent and opaque morphological primes (e.g., Rastle et al., 2004; Longtin et al., 2003; Marelli et al., 2013), these primes yielded no more facilitation than orthographic controls for L2 readers.

These reports of a different morphological priming profile in L2 were not confirmed in Diependaele et al. (2011). These authors tested two groups of Dutch and Spanish non–native speakers of English in a masked priming study, with the same conditions that were typically adopted in the vast L1 literature – transparent suffixed primes (e.g., *viewer–VIEW*) were contrasted with opaque (pseudo–)suffixed primes (e.g., *corner–CORN*) and orthographically–matched, non–morphological controls (e.g., *dialog–DIAL*). They reported no statistically significant difference between morphological priming in L1 and L2, contrary to Silva and Clahsen (2008). Across three experiments, they seem to observe a graded facilitation pattern where genuine morphological priming is larger than the morpho–orthographic effect, which in turn exceeds orthographic priming. However, individual experiment data were not clear cut. Opaque priming, for example, did not differ statistically from the orthographic baseline in any of the three individual studies, making the genuine contribution of morphology somewhat unclear.

One aspect in which the studies described above agree is, interestingly, outside of their main scope. Namely, they report more orthographic priming (e.g., *colonel–COLON*) in L2 than in L1, and no priming at all in this latter. This pattern is confirmed in Heyer and Clahsen (2015), where transparent, derivational priming was only contrasted with the orthographic effect – opaque primes were not part of the design. In their masked condition, these authors report the standard pattern in L1, with significant morphological facilitation and no orthographic effect. In L2, instead, this latter was equal to morphological priming.

The interaction of L1–L2 with form priming did not receive much attention because in all these studies the orthographic condition was effectively a control baseline. Diependaele et al. (2011) explain it in terms of slower prime processing. Here, we offer a more intriguing interpretation, which relates to the literature on novel word learning and form priming in L1. It is well established that nonword primes (e.g., contraft–CONTRACT) yield larger orthographic facilitation than word primes (e.g., contrast–CONTRACT) in L1, a phenomenon known as Prime Lexicality Effect (PLE; e.g., Forster and Veres, 1998). PLE is typically interpreted in terms of lexical competition – with word primes, the gain that one gets from shared letters is offset by the competition in the lexicon between the prime and the target representations. Lexical competition, in turn, has been often taken as a benchmark for the consolidation of new lexical memories (Gaskell and Dumay, 2003; Tamminen and Gaskell, 2008; Davis and Lupker, 2006). Therefore, orthographic priming from real words in L2 could be attributed to a still–incomplete consolidation process, whereby memories for novel words are perhaps present in the brain, but not yet fully lexicalised. Essentially, they would work similarly to nonwords in L1; because they do not participate in lexical competition, they yield priming based on sub–lexical processing. This interpretation connects nicely with recent evidence in L1, showing that prime lexicality modulates morphological facilitation in French native speakers (Grainger and Beyersmann, 2020). Moreover, it connects to general theories of lexical and language learning (Ullman, 2001, 2005). In this paper we attempt to provide a fuller description of L2 morphological priming. We do this by replicating this orthographic effect described above, qualifying it through consideration of individual variability (see below), and developing its theoretical implications more fully.

Morphological priming in L2 has also been studied with inflected primes; however, the picture remains difficult to interpret. Kirkici and Clahsen (2013) compared the processing of inflected and derived words in non–native speakers of Turkish using a series of masked priming experiments. The non–native speakers involved in this study had a variety of L1 backgrounds, but were all highly proficient. Priming in L1 turned out to be equivalent for inflection (*sorar–SOR*, s/he asks–ASK) and derivation (*yorgunluk-YORGUN*, tiredness–TIRED). In L2 instead, derivational priming was larger than the inflectional effect, which did not emerge at all.

In an experiment with regular (e.g., *billed-BILL*) and irregular (e.g., *fell-FALL*) masked primes, Feldman et al. (2010) reported statistically different patterns of facilitation in L1 and L2 speakers of English. The critical interaction between regularity and prime relatedness was not significant in L2, although some further post-hoc analyses revealed that regular inflected primes, but not irregular inflections, did provide facilitation as compared to an orthographic baseline (e.g., *billion-BILL, fill-FALL*). In line with the data from derivational priming (e.g., Diependaele et al. 2011), form priming was significant in L2. Coughlin and Tremblay (2014) assessed verb inflectional priming in French with a similar design: They compared morphological primes (e.g., *donnons-DONNE, (we) give- (I) GIVE*) to both an orthographic (e.g., *doute-DONNE, (I) doubt- (I) GIVE*) and an unrelated baseline (e.g., *parle-DONNE, (I) speak- (I) GIVE*). In this study, the pattern of facilitation did not differ statistically in native and non-native speakers, contrary to Feldman et al. (2010). Similarly to Feldman et al.’s results, orthographic priming was again significant in L2; however, contrary to a vast body of literature (e.g., Forster and Veres, 1998; Davis and Lupker, 2006), this was also the case for L1. Further data from a phrase completion experiment suggests that noun inflection differs in L1 vs. L2 speakers (Foote, 2015).

Why does morphological priming prove to be so difficult to characterize in L2? A strong candidate to explain inconsistency in the previous data is surely individual variability. Evidence is accumulating that visual word identification is heavily influenced by the individual profile of each reader (e.g., Andrews and Hersch, 2010; Andrews and Lo, 2013; Milin et al., 2017). In a masked derivational priming experiment, Andrews and Lo (2013) showed that the pattern of facilitation between transparent, opaque, and orthographic primes changes as a function of readers’ spelling skills (as compared to their vocabulary). They report that priming is very similar in the opaque and transparent conditions when spelling skills are strong, but when spelling skills are weak, opaque priming is less pronounced and may even be equivalent to the orthographic baseline. Milin et al. (2017) also provided data suggesting that some readers may not show a difference between opaque and form priming. Along similar lines, Beyersmann et al. (2015a) reported that individuals with high vocabulary and spelling competence display facilitation from non–suffixed nonword primes (e.g., bankord-BANK), while readers with relatively weaker skills do this to a much lesser extent. They take these results to show that morphological processing depends on more general lexical and orthographic skills: People with relatively lower levels of language proficiency rely more heavily on morphological segmentation than individuals with relatively higher levels of language proficiency (see also Grainger and Beyersmann, 2017). Similar conclusions have been drawn in studies using a combination of vocabulary and spelling abilities to index individual differences in morphological priming among novice readers (Beyersmann et al., 2015b; Hasenäcker et al., 2015).

All these results relate to L1, and it is not obvious that they generalise to L2. Conversely, these effects may even be magnified in L2, where inter–subject variability is likely enhanced by the diversity of the learning experience. Factors like Age of Acquisition (AoA) or proficiency may well mean different cognitive processes are in place when L2 readers are exposed to printed words. Along these lines, Dawson et al. (2017) have recently shown that morphologically structured nonwords (e.g., earist) are more likely to be taken as words than control stimuli (e.g., earilt) in adults and adolescents, but not in younger children. These data refer to L1, but do show that less experience with printed words may determine a different morphological processing – this may apply to L2 speakers as well as children learning L1 (see also Beyersmann et al., 2015a, b; Grainger and Beyersmann, 2017). Another recent study by Veríssimo et al. (2018) investigated masked morphological priming in Turkish–German bilinguals, and found an effect of AoA on inflectional, but not derivational L2 priming, suggesting that sensitivity to morphological features is constrained by the learning trajectory of a (second) language.

Most previous studies on L2 derivational priming did not try to characterize their participants’ profile in terms of proficiency or AoA beyond self reports, nor to investigate whether and how these individual features may modulate the priming pattern. An exception is perhaps Li et al. (2017), which reports on a masked morphological priming experiment with Chinese-English bilinguals. The separation of participants into high-proficiency and low-proficiency groups was validated via a questionnaire on English usage and a vocabulary test (although neither metric was then used directly to model priming). Proficiency was found to interact with morphological facilitation; lower-proficiency L2 readers showed significant priming in the transparent and form conditions, but not in the opaque condition, while more proficient participants showed the predominant L1 pattern, with significant transparent and opaque priming, but no form priming. Working with inflectional primes, Feldman et al. (2010) obtained somewhat different results. Proficiency did not interact significantly with the priming pattern in the main statistical model. However, the authors carried out separate analyses for higher and lower-proficiency readers. They found genuine sensitivity to morphology, similarly to L1, only for the former group; in lower-proficiency participants, morphological primes did not yield any additional advantage as compared to an orthographic baseline. In contrast, Coughlin and Tremblay (2014) observed that the difference between morphological and orthographic priming does not interact with readers’ proficiency, nor does the comparison between orthographic and unrelated primes, i.e., form priming *per se*.^2^ So, overall the pattern is far from clear.

It is important to note that proficiency was defined somewhat coarsely in these studies, based on a questionnaire on language usage and a vocabulary test (Li et al., 2017), on speed and accuracy in the priming task itself (Feldman et al., 2010), or on a single sentence completion task (Coughlin et al., 2019). Although these are legitimate approximations, language competence is a highly multicomponent construct and can be assessed more widely. In the present study, we probe our participants’ L2 proficiency with a battery of tests covering seven language domains (morphological awareness, fluency, phonemic discrimination, vocabulary, spelling, oral and reading comprehension). We also assess AoA (and, more generally, the participants’ learning experience) through a questionnaire. Most importantly, we explicitly tried to recruit readers with varying learning experiences and proficiency, so that we could properly assess whether L2 morphological priming is affected by these factors.

Another recent development in the literature is the discovery that readers’ morphological processing is mediated by their sensitivity to graded, probabilistic relationships between form and meaning. Marelli et al. (2015) quantified these relationships in terms of what they called Orthography–to–Semantics Consistency (OSC) – a frequency–weighted average of the semantic similarity between all members of a given morpho–orthographic family and their stem. Consider, for example, the word *corn*. If we take all the words in the lexicon that start with the string *corn* – that is, that might potentially have *corn* as a stem – we obtain items like *corny, cornish* or *corner*. Because these words are fairly unrelated in meaning with *corn*, OSC would be low. By contrast, a stem like *risk*, whose morpho-orthographic neighbourhood would be populated by words like *risks, risked*, or *risky*, would have higher OSC, given that all these words are genuine morphological relatives and are therefore strongly related in meaning. In a large scale regression analysis, Marelli et al. (2015) showed that words with higher OSC (that is, words that are part of semantically consistent morpho-orthographic families) are identified more quickly.

More recently, Amenta et al. (2020) showed that OSC modulates morphological priming specifically. This is particularly interesting because OSC is a property of the target itself, independently of any specific prime: *cornfield-CORN* would have the same exact OSC of *cornice-CORN*, while, for example, the former pair would be considered more semantically transparent than the latter. So, Amenta et al.’s results highlight the importance of the lexical-semantic region target words of priming experiments live in – more specifically, the consistency of the mapping between form and meaning there. In a sense, this extends the scope of semantic transparency well beyond the specific relationship between a target and any given prime: this factor is surely important in itself, but it is also qualified by the entire set of the target’s morpho-orthographic neighbours, independently of which of these neighbours was actually used as a prime on any given instance. More generally, the pattern of results described in Amenta et al. (2020) might be taken to downplay the role of discrete categories, which may have given rise to inconsistent results at times (e.g., Davis, 2010; Feldman et al., 2009, 2012) and have proven difficult to define precisely in some instances (e.g., the word *fruitless* is not directly related to the literal meaning of *fruit*, but there is a metaphorical sense where the stem is more transparent, and the suffix is quite transparent, too; Baayen et al., 2011). In this novel view, priming would be modulated by a network of probabilistic ties between form and meaning, which potentially extends beyond morphology *per se* (e.g., phonaestemes; Baayen et al., 2011; Marelli et al., 2015). Classic concepts like segmentation or affix/stem identification might also fade to the background, and the debate on the role of semantics in early processing would take an important turn: An effect of OSC does require an early access to semantic information, but also implies that any potential orthographic unit (including pseudo-affixes in opaque words) is activated independently of whether it will actually turn out to be semantically transparent (Amenta et al., 2015).

Of course, the appreciation of these fine–grained ties between form and meaning likely requires a rather extensive experience with any lexicon. Thus, one can imagine that L2 speakers would show less sensitivity to OSC; or perhaps more intriguingly, that their sensitivity grows with proficiency. Or perhaps again, one needs early exposure to a language in order to see a probabilistic form–meaning relationship structure, so that only early–AoA participants would show an effect of OSC. More generally, OSC offers an interesting perspective on the learning of a second language, which may involve a growing sensitivity to probabilistic ties between form and meaning. We will try to shed light on this issue by checking whether morphological priming – and, more generally, word identification time – is modulated by OSC in L2.

To summarise, the present experiment tries to clarify how bilingual readers process word morphological structure in L2, primarily by characterizing their profile in terms of proficiency and age of acquisition. Moreover, we will check how and whether fine–grained, probabilistic relationships between form and meaning (as tracked by Orthography–to–Semantics Consistency) inform L2 visual word identification, which speaks to the hypothesis that learning a novel lexicon proceeds through an increased appreciation of the statistical structure of the orthography–semantics mapping.

## 2 Methods

### Participants

81 students at the University of Trieste participated in the study. They were 73 right–handed and 8 left–handed native speakers of Italian, who provided informed written consent to take part into the experiment. Their mean age was 24.3 years (range: 18–34) and their mean education was 17 years (range: 13–22); 27 of them were males. Participants had no history of neurological impairment or learning disabilities, and normal or corrected-to-normal vision. They were compensated for their time with 20 Euros. All participants took part in both the Italian–L1 and the English–L2 masked priming experiments.

### Materials

The Italian set of stimuli is composed of 150 prime–target pairs, 50 in each of three conditions. Primes and targets in the *transparent* condition entertain a genuine morphological relationship (e.g., *artista–ARTE*, artist–ART). Primes and targets in the *opaque* condition are semantically independent, but entertain an apparent morphological relationship, i.e., primes are made of a pseudo-stem, which is shared with the targets, and a pseudo-suffix (e.g., *retaggio–RETE*, legacy–NET; an analogous example in English would be corner–CORN). Primes and targets in the *form* condition have a purely orthographic relationship, i.e., primes share a (pseudo-)stem with their targets, but end in a non–suffix (e.g., *corallo–CORO*, coral–CHOIR; an analogous example in English would be dialog–DIAL). Targets and primes were matched across condition for frequency (as indexed by the SUBTLEX–IT database; Crepaldi et al., 2013), length, Coltheart’s N and prime–target orthographic similarity (see Table 1).

**Table 1:** Stimulus statistics for the Italian L1 set; we report means and standard deviations. Frequency is reported in Zipf (Brysbaert et al., 2018).


	TRANSPARENT	OPAQUE	ORTHOGRAPHIC

Target frequency	3.96 (0.67)	3.63 (0.87)	3.94 (0.84)

Target length	5.16 (1.07)	5.08 (0.84)	4.94 (0.88)

Target Coltheart’s N	18.1 (11.3)	20.1 (11.9)	21.5 (13.4)

Related prime frequency	2.92 (0.84)	3.15 (0.78)	3.22 (0.69)

Control prime frequency	2.91 (0.68)	3.09 (0.85)	3.19 (0.67)

Related prime length	7.70 (1.24)	7.96 (1.21)	7.52 (1.18)

Control prime length	7.70 (1.24)	7.96 (1.21)	7.52 (1.18)

Related prime Coltheart’s N	3.6 (2.9)	3.5 (2.6)	4.2 (6.1)

Control prime Coltheart’s N	3.8 (2.9)	3.8 (2.9)	3.5 (2.5)


For each related prime, we selected a control prime that is semantically, orthographically, and morphologically unrelated to the targets (e.g., *plunder–ACRE*). Control primes were matched as closely as possible to related primes on frequency, length and Coltheart’s N (see Table 1). In order to avoid multiple presentations of the same target word to the same participant, we rotated related and control primes over two lists, in a Latin Square design; thus, each participant saw each target, either paired with its related or control prime.

150 nonword targets were also selected to serve as NO trials in the lexical decision task. They were matched with word targets on length (mean = 5.06, SD = 0.95). Each of these targets was paired with a word prime, mirroring the structure of the word target set: Half of these primes were orthographically similar to their targets, and *2/3* of the primes were complex words. This served the purpose of leaving the primes devoid of any information about the lexicality of their targets. These prime words were also roughly matched with the word–target primes for frequency (mean = 3.18, SD = 0.87), length (mean = 7.42, SD = 1.32), and Coltheart’s N (mean = 3.02, SD = 3.5).

The English set of stimuli perfectly mirrors the Italian one. It is largely based on Rastle et al. (2004), with only a few additions and replacements. The lexical statistics of these stimuli are reported in Table 2. Frequency values are based on SUBTLEX–UK (Van Heuven et al., 2014).

**Table 2:** Stimulus statistics for the English L2 set; we report means and standard deviations. Frequency is reported in Zipf (Brysbaert et al., 2018).


	TRANSPARENT	OPAQUE	ORTHOGRAPHIC

Target frequency	4.09 (0.72)	3.88 (0.74)	3.72 (0.82)

Target length	4.92 (0.65)	4.80 (0.69)	4.62 (0.68)

Target Coltheart’s N	6.66 (5.73)	9.08 (7.78)	11.72 (8.2)

Related prime frequency	3.32 (0.93)	3.43 (0.96)	3.50 (0.93)

Control prime frequency	3.30 (0.83)	3.46 (1.03)	3.47 (0.87)

Related prime length	7.12 (1.15)	7.09 (1.19)	7.15 (1.68)

Control prime length	7.12 (1.11)	7.09 (1.16)	7.15 (1.67)

Related prime Coltheart’s N	1.98 (2.5)	2.50 (2.9)	2.06 (3.2)

Control prime Coltheart’s N	3.4 (4.6)	2.64 (4.7)	3.04 (4.2)


The complete list of Italian and English stimuli is offered in the Appendix.

### Measures of proficiency in English

English L2 proficiency was assessed via a battery of tests that cover phonemic fluency, phonemic discrimination, spelling, vocabulary, morphological awareness, and oral and reading comprehension.

*Phonemic fluency*. Participants were asked to produce as many words as possible starting with the phonemes /f/ or /p/, in two separate 60–second sessions. Answers were recorded through a microphone for off–line scoring. Each participant’s score is the total number of words produced.

*Phonemic discrimination*. Participants were acoustically presented with a probe pseudo–word (e.g., *kneef*), and then with three test pseudo–words (e.g., *yawk, zeep, wid*). They were asked to pick up which of the test pseudo–words shared one phoneme with the probe. The score is the number of correctly identified test pseudo–words, out of the 13 trials that made up the task. The shared phoneme could be either a consonant or a vowel.

*Spelling*. 20 words were recorded by a native speaker of English, and included in example sentences to clarify any lexical ambiguity. These words were then presented to the participants, who were required to write them. Words were selected from Burt and Tate (2002), among those that were correctly spelled by between 30% and 90% of a sample of Australian first–year university students. This test is taken from Andrews and Lo (2013), with Latin derivations excluded because Italian speakers may be able to reconstruct their spelling based on etymology. Participants’ score for the test was the number of correctly spelled words.

*Vocabulary*. This task comes from the Test of English as a Foreign Language (TOEFL), and consists of 20 sentences presented in a written form that are completed by choosing a proper word among three alternative choices. The score for this test is the number of correct choices.

*Morphological awareness*. This test was presented in a written form, and consisted of 9 sentences that participants were asked to fill with an appropriate plausible pseudo–word, chosen among two options. Nonwords contained a suffix, which unambiguously made only one option a plausible sentence completion (e.g., *The tiny coral snake is ____ (valgeful/valgefully) but deadly*). The score for the test is the number of correct picks.

*Oral comprehension*. This test also comes from the TOEFL. Participants listened to two conversations between English native speakers, and were then asked 6 comprehension questions about them. They marked the correct answer among 4 alternatives. The score for the test is the number of correct answers.

*Reading comprehension*. Participants were required to read a text passage of approximately one page, and answer some comprehension questions. This task was taken again from the TOEFL, and consisted of seven questions, each with 4 alternative choices. The score is again the number of correct answers.

### Measures of Age of Acquisition of English

Age of Acquisition of English (henceforth, AoA) was assessed via a questionnaire, which we expanded to include items on perceived proficiency and language experience in general. The questionnaire was composed of the following questions:

1. Which age were you exposed to English for the first time? (AoA proper)
2. Indicate how much you use English in your daily life from one (never) to five (always)
3. In which context were you exposed to English for the first time – home or school?
4. Did you grow up in a context where multiple languages were spoken?
5. How would you rate your proficiency in English, from 1 (very bad) to 5 (very good)?
6. Do you speak any other languages in addition to Italian and English?

### Procedure

Participants completed the AoA questionnaire online through the Department’s participant recruitment system. The rest of the data collection happened in the lab, in two sessions. During the first session, which lasted around an hour, participants carried out the proficiency tests. During the second session, participants underwent the lexical decision experiment, both in Italian (L1) and English (L2). This session lasted around 40 minutes. The testing order for the two languages was counterbalanced across participants.

For the lexical decision task, participants were tested in a soundproof, dimly lit booth. Stimuli were presented in a randomized order using Psychopy2 (Peirce et al., 2019), and responses were collected through a two–button, custom–made response box based on Arduino microcontroller boards (https://www.arduino.cc/). The YES button was always controlled by the dominant hand.

Each trial started with a string of hash marks, presented for 500 ms, which was replaced by the prime, presented for 50 ms in lowercase. The prime was immediately followed by the target, presented in uppercase until response, or for 2000 ms. All stimuli were presented in the center of the screen.

Participants were not informed of the presence of the prime, and were asked to respond as quickly and as accurately as possible. Twelve practice trials preceded the “proper experiment”, to allow familiarization with the task. At the end of the session, participants were debriefed to check whether they noticed the presence of a prime.

### Statistical Analysis

Response time analyses were carried out on correct trials only. Exclusions were applied separately for the Italian (L1) and English (L2) datasets.^3^ For Italian, we excluded one participant who was aware of the primes; two participants whose accuracy on nonwords was below 80%. We also excluded all trials concerning three target words, which were responded to correctly less than 60% of the time over all participants, and individual data points below 280 ms or above 2500 ms. This resulted in the exclusion of 526 datapoints, which amounts to 4.6% of all available data. We were then left with 11009 data points for the analysis.

In the English set, we excluded two participants who reported having seen the primes; one additional participant whose mean overall response time was under 200 ms; and individual data points that were below 300 ms or above 2000 ms. This led to the exclusion of 281 datapoints, which is 3% of the total available data. The clean dataset was comprised of 8938 data points. There were fewer English data points than Italian data points because participants made more mistakes on English trials than Italian trials.

Generalized linear mixed models were used to fit reaction times within the R environment (R Development Core Team, 2008), using the package lme4 (Bates et al., 2015). We resorted to GLMMs in order to avoid RT transformations, which have been shown to potentially distort the data pattern (for an extensive discussion about this topic, see Balota et al., 2013; Lo and Andrews, 2015). Following Lo and Andrews (2015), we adopted a Gamma distribution with an identity link function.^4^ The effects of interest were *prime relatedness* (related vs. unrelated), *morphological type* (transparent vs. opaque vs. orthographic), and their interaction. For the proficiency and AoA analyses, we added each individual predictor tracking these variables (i.e., each test score and each questionnaire item) to the main interaction, one at a time to avoid excessive collinearity. Trial position in the randomised list, target frequency, target length, target orthographic neighborhood size and rotation were also added as fixed effects, to control for spurious variance. In general, only those variables that produced a significant increase in goodness of fit were retained in the analyses, as determined via the anova function comparing hierarchical models. The statistical significance of the effects of interest was assessed using Type III sum-of-squares and *χ^2^* Wald tests as implemented in the Anova function from the car package (Fox and Weisberg, 2019). When this test was significant, we explored the model parameters via the anova function and computed model-based response time estimates through the package effects (Fox and Hong, 2009). All figures were created using ggplot2 (Wickham, 2016).

### Data availability and open science

All data, stimuli and code that were used in the context of this experiment are openly available at the Open Science Framework.

## 3. Results

**Figure 1:**
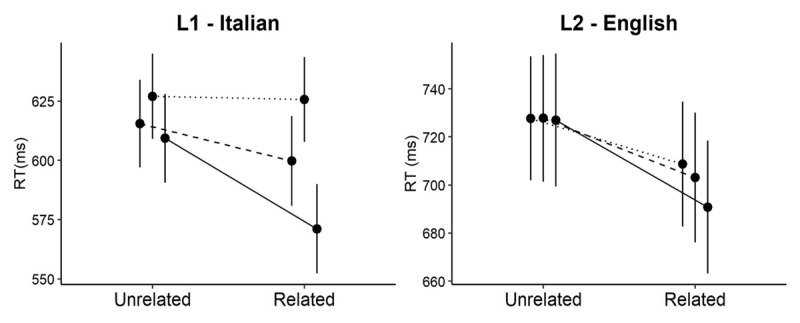
Model–based estimates of response times per condition, in L1 (left panel) and L2 (right panel). The solid, dashed and dotted lines represent the transparent, opaque and orthographic conditions, respectively. Error bars are 95% confidence intervals.

Figure 1 depicts the model-based average estimates of response times given by participants per condition (i.e., transparent, opaque and orthographic) in each language (L1; Italian - L2; English). The overall mean RT and accuracy in the task were 594 ms and 95% respectively, for Italian; and 672 ms and 76% for English (81% in the transparent condition, 75% in the opaque condition and 70% in the orthographic condition).

The model for the Italian data reveals a significant interaction between *prime relatedness* and *morphological type, χ^2^*[2] = 228.8*, p <* .001. The interaction is driven by significantly more priming in the transparent, *β* = –36.8*, t* = –14.5*, p <* .0001, and opaque conditions, *β* = –14.2*, t* = –6.4*, p <* .0001, as contrasted with the orthographic condition, which does not seem to show any facilitation, *β* = –1.3*, t* = –0.64*, p* = .52. Transparent priming is also significantly larger than opaque priming, *β* = –22.5*, t* = –6.8*, p <* .0001.

The model for the English data also reveals a significant interaction between *prime relatedness* and *morphological type, χ^2^*[2] = 32.9*, p <* .0001. Similarly to Italian, transparent primes yield more facilitation than orthographic primes, *β* = –17.1*, t* = –5.5*, p <* .0001, thus confirming that L2 speakers are fully sensitive to genuine, semantically transparent morphology. The difference between opaque and orthographic priming is only marginally significant, *β* = –5.6*, t* = –1.9*, p* = .058, probably due to the most glaring difference between the English and the Italian data – orthographic primes yield significant facilitation themselves, *β* = –19*, t* = –5.34*, p* = .0002. Finally, transparent primes seem to provide larger priming than opaque primes, *β* = –11.5*, t* = –3.7*, p* = .0002.

A cross–language analysis confirms that the priming pattern across conditions is different in L1 and L2, as attested by the significant interaction between *prime relatedness, morphological type*, and *language, χ^2^*[2] = 61.7*, p <* .0001.^5^

### Language proficiency and priming

**Figure 2:**
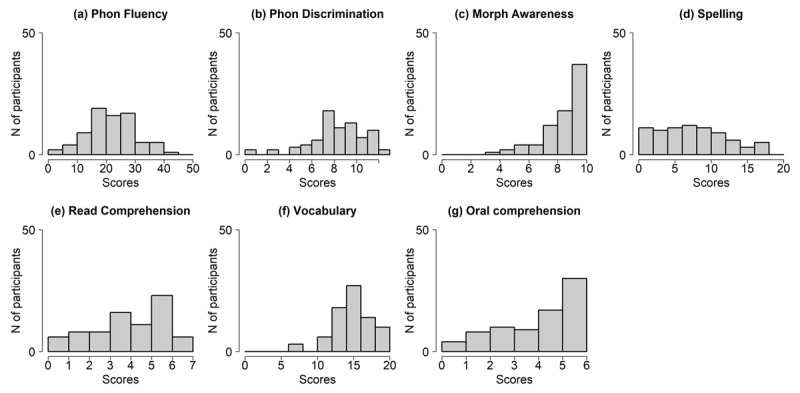
Participants’ score distributions for each English proficiency subtest.

**Figure 3:**
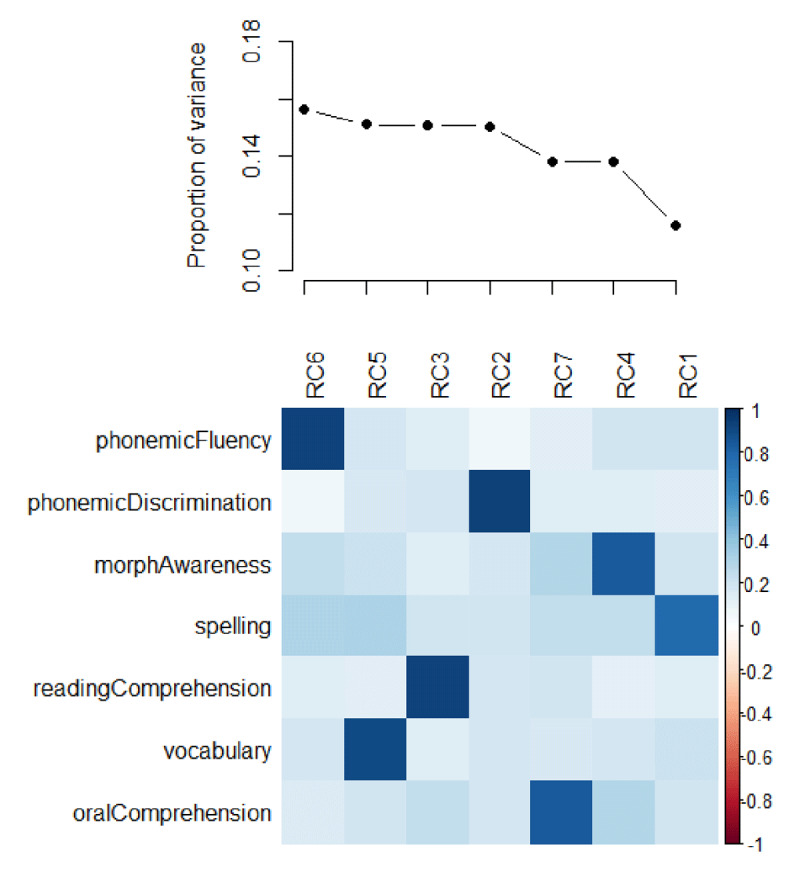
Illustration of the Varimax Principal Component Analysis on the seven proficiency metrics. The upper panel reports on the amount of variance accounted for by each Principal Component (RC). The lower panel describes the correlation between each Principal Component and the seven proficiency metrics; color codes for the strength of the correlation, as illustrated by the colorbar on the right.

L2 proficiency scores are distributed as illustrated in Figure 2 – we were able to sample a rather wide distribution of proficiency across different linguistic domains. The correlation between pairs of indices is reported in Table 3, and varies between *.25* and .68 (lower quartile = .43, median = .46, upper quartile = .54). This attests the effectiveness of the battery – individual scores correlate enough to be credible measure of individuals’ proficiency, but also vary enough to effectively track different aspects of L2 competence. Note, however, that these observed correlations likely underestimate the true correlations in the population, as long as the tests that we used do not have perfect test-retest reliability (Spearman, 1904) – which is very likely to be the case, as for any psychometric test. Spelling engages in particularly strong correlations (*r >* .60), with phonemic fluency, morphological awareness, vocabulary and oral comprehension. Morphological awareness and oral comprehension also correlate quite strongly (*r* = .68). To explore more in depth the structure underlying these correlations, we ran a Principal Component Analysis (PCA) using a Varimax rotation. The results are illustrated in Figure 3 and indicate that (i) seven Principal Components are necessary to account for this set of correlations, i.e., they all explain a similar and substantial amount of variance; and (ii) each of them map clearly to one specific proficiency metric. This suggests that the seven variables we considered here constitute a minimal set of interpretable predictors; we could obviously drop some of these metrics, but we would lose independent (and potentially important) information. The PCA thus provides strong validation to the battery of tests that we adopted.

**Table 3:** Correlation among the English proficiency subtests.


	PHON-FLUENCY	PHONDISCRIMINATION	MORPH-AWARENESS	SPELLING

phonFluency	1	0.25	0.54	0.61

phonDiscrimination	0.25	1	0.43	0.46

morphAwareness	0.54	0.43	1	0.64

spelling	0.61	0.46	0.64	1

readComprehension	0.35	0.44	0.4	0.49

vocabulary	0.45	0.44	0.54	0.65

oralComprehension	0.43	0.45	0.68	0.62

	**READ-COMPREHENSION**	**VOCABULARY**	**ORAL-COMPREHENSION**	

phonFluency	0.35	0.45	0.43	

phonDiscrimination	0.44	0.44	0.45	

morphAwareness	0.4	0.54	0.68	

spelling	0.49	0.65	0.62	

readingComprehension	1	0.37	0.53	

vocabulary	0.37	1	0.51	

oralComprehension	0.53	0.51	1	


As illustrated in Figure 3 above, we assessed the impact of each subtest on L2 priming in a separate model to avoid excessive collinearity. In fact, the condition number *K* (Belsley, 1980) is 33.92–, above the threshold of 30 that indicates harmful collinearity if we were to use all predictors in one unique model (Baayen et al., 2008). Note that, because we did not have strong predictions as to which specific proficiency metric might work best, we did not focus on any specific measure and adopted a rather exploratory approach.

Every individual sub-test improves overall goodness of fit, *χ^2^*[6] = 15.79–35.02, all p values <.014 – quite unsurprisingly, RTs are better accounted for when participants’ proficiency is taken into account. This improved goodness of fit does not necessarily come from morphological priming modulation; proficiency might just explain overall response speed, or general sensitivity to priming. We thus assessed which proficiency score, if any, interacted specifically with prime relatedness and morphological condition. This happens for phonemic discrimination, *χ^2^*[2] = 42.5*, p <* .0001, vocabulary *χ^2^*[2] = 39.3*, p* = .001, and morphological awareness *χ^2^*[2] = 16.3*, p* = .0002. The remaining tests – phonemic fluency, spelling, oral comprehension and reading comprehension – did not reach significance (all *χ^2^*s < 4.78, all *p*s > .09).^6^

**Figure 4:**
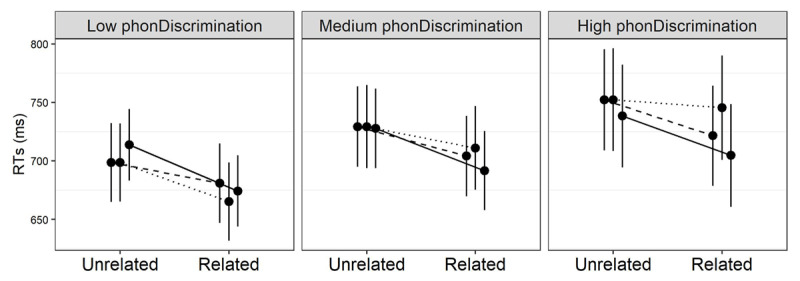
Model–based estimates of response times (RTs) relative to the interaction between prime relatedness, morphological type, and phonemic discrimination in L2. The solid, dashed and dotted lines represent the transparent, opaque and orthographic conditions, respectively. Effects are estimated at the 5th, 50th (median) and 95th percentile of the phonemic discrimination distribution. Error bars are 95% confidence intervals.

For phonemic discrimination, the nature of the priming modulation is illustrated through the model–based estimates in Figure 4. Transparent and opaque priming seem to be solid and consistent across the whole phonemic discrimination spectrum, while orthographic priming appears to shrink with growing performance. This is supported by the model parameters, where orthographic priming is significantly different from both opaque, *β* = –5.65*, t* = –6.45*, p <* .0001, and transparent priming, *β* = –2.94*, t* = –3.45*, p* = .0006, while the latter two conditions do not differ, *β* = .55*, t* = –.75*, p* = .45.

**Figure 5:**
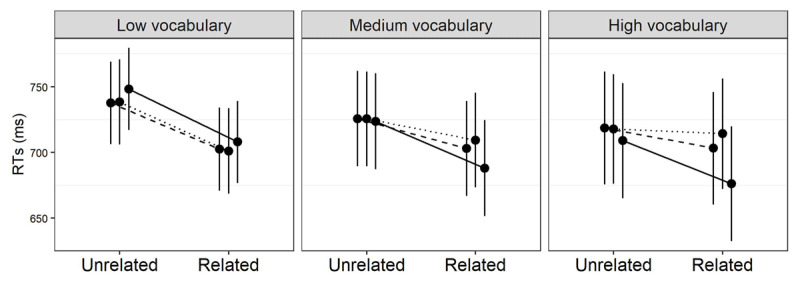
Model–based estimates of response times (RTs) relative to the interaction between prime relatedness, morphological type, and vocabulary in L2. The solid, dashed and dotted lines represent the transparent, opaque and orthographic conditions, respectively. Effects are estimated at the 5th, 50th (median) and 95th percentile of the vocabulary distribution. Error bars are 95% confidence intervals.

The priming modulation pattern is similar for vocabulary (Figure 5). Transparent facilitation is again consistent across different levels of vocabulary skills, while orthographic priming shrinks significantly with growing proficiency, *β* = –3.28*, t* = –6.21*, p* = .001. Again similarly to the phonemic discrimination results, opaque priming differs from orthographic priming, *β* = –1.75*, t* = –3.31*, p* = .0009. Unlike the phonemic discrimination results, however, opaque priming also differs from transparent facilitation, *β* = –2.32*, t* = –3.73*, p* = .0001.

So, transparent priming is consistently strong and independent of L2 proficiency, whereas form priming consistently shrinks towards zero with growing proficiency. Opaque priming resembles the transparent condition in the phonemic discrimination analysis, while it differs from transparent priming in the vocabulary results.

**Figure 6:**
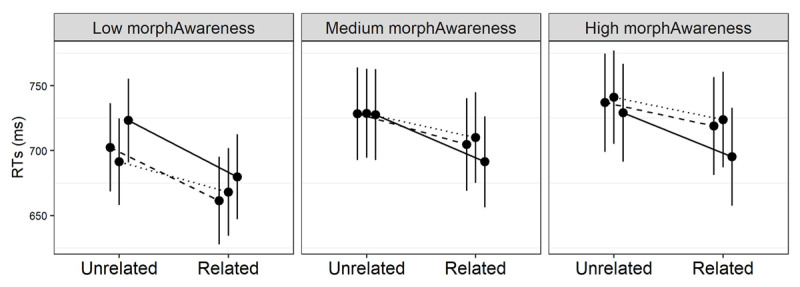
Model–based estimates of response times (RTs) relative to the interaction between prime relatedness, morphological type, and morphological awareness in L2. The solid, dashed and dotted lines represent the transparent, opaque and orthographic conditions, respectively. Effects are estimated at the 5th, 50th (median) and 95th percentile of the morphological awareness distribution. Error bars are 95% confidence intervals.

The pattern for morphological awareness (Figure 6) shows again that transparent priming remains strong across the board. Contrary to the previous metrics, however, so does form priming, which is not significantly different from the transparent condition, *β* = .084*, t* = 0.97*, p* = .32. The odd one out is now opaque priming, which shrinks with growing morphological awareness more than both the form, *β* = –4.23*, t* = 4.03*, p <* .0001, and the transparent condition, *β* = –5.58*, t* = –6.58*, p <* .001.

**Figure 7:**
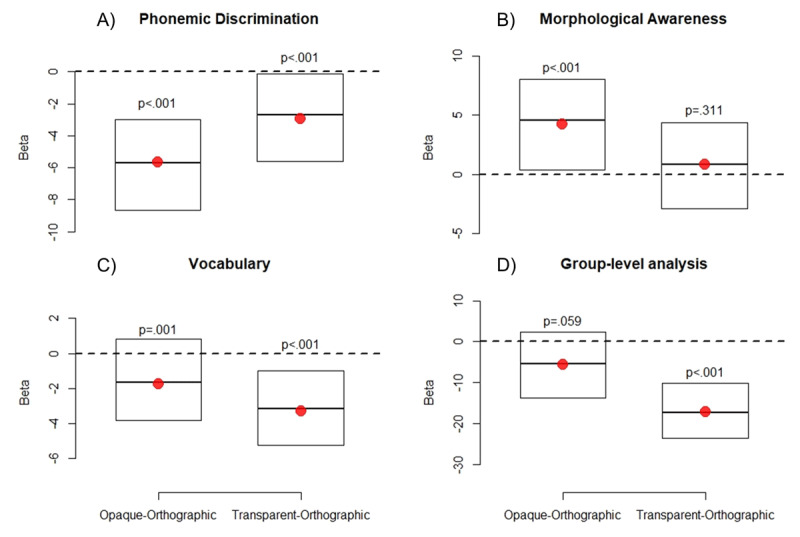
Jackknife results on the proficiency analysis. We used 200 replicates and, on each replicate, selected 40 out of 50 targets per condition, per participant. Each median estimate (the bold lines in the graphs) matches perfectly the full model estimates (the red dots). Also, the 5th and 95th percentiles (which define the boxes in the graphs) reflect nicely the significance of the estimated parameters in the full model (which is reported jut above the boxes as a p value). Panel (a), (b) and (c) refer to the proficiency metrics that turned out to modulate priming in L2, while panel (d) refers to the L2 group-level analysis, for comparison.

Since error rates were relatively high in L2, which reduced the number of datapoints available to the models, we further assessed the reliability of the proficiency results via a *jackknife* procedure (Ang, 1998) – we repeatedly fitted the models described above to a subsample of the original observation set and checked that the model estimates remained fairly stable. These additional analyses fully confirm the pattern of results illustrated above, as shown in Figure 7.

### AoA analysis

**Figure 8:**
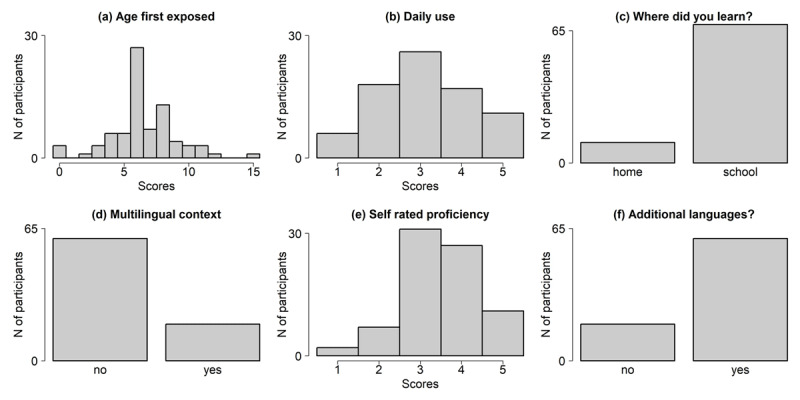
Scores distributions in the AoA questionnaire. All participants were Italian native speakers, the questions refer to English as a second language.

The scores collected through the AoA questionnaire on L2 are distributed as illustrated in Figure 8. AoA proper, panel (a), is reasonably well distributed, with a peak around the age of 6, which is the age children enter school in Italy. This coheres with the fact that most of our participants learned English at school, panel (c). Interestingly, we also happened to recruit few participants with *AoA<6*, who learned English at home. Quite notable are the nicely symmetrical distributions for daily use of English, panel (b), and self–rated proficiency, panel (e). Finally, most of our participants did not grow up in a multilingual environment, panel (d), but ended up speaking at least another language in addition to Italian and English, panel (f). This is expected considering the rich multiculturalism of Trieste. Importantly, AoA proper correlates *–.15* with daily usage, *.04* with self rated proficiency, and never stronger than |.23*|* with the objective proficiency scores; this means that we can assess the effect of AoA independently of other variables.

We followed the same modelling approach as for proficiency, that is, we first assessed whether AoA proper allows an overall better account of RTs. This does not seem to be the case, *χ^2^*[6] = 10.61*, p* = .10, which suggests that age of acquisition does not contribute to explaining morphological priming data.

Among the other scores that we collected via the AoA questionnaire, only self-rated proficiency improves the quality of the model predictions, *χ^2^*[6] = 17.78*, p* = .006. Self-rated proficiency also modulates L2 priming, *χ^2^*[2] = 68.08*, p <* .001, thus nicely confirming the pattern revealed by the objective proficiency scores. Self-rated proficiency perfectly mirrors the phonemic discrimination results illustrated above: orthographic priming shrinks with growing proficiency significantly more than both opaque, *β* = –13.45*, t* = –6.46*, p <* .0001, and transparent priming, *β* = –13.77*, t* = –6.95*, p <* .0001, while there is no difference between the latter two *β* = –.3*, t* = –0.16*, p* = .86.

The remaining four variables (speaking a third language, learning L2 at school vs. home, and learning L2 in a multilingual environment) do not affect RTs, all *χ^2^*[6] < 5.87, all *p >* .48.

### OSC analysis

**Figure 9:**
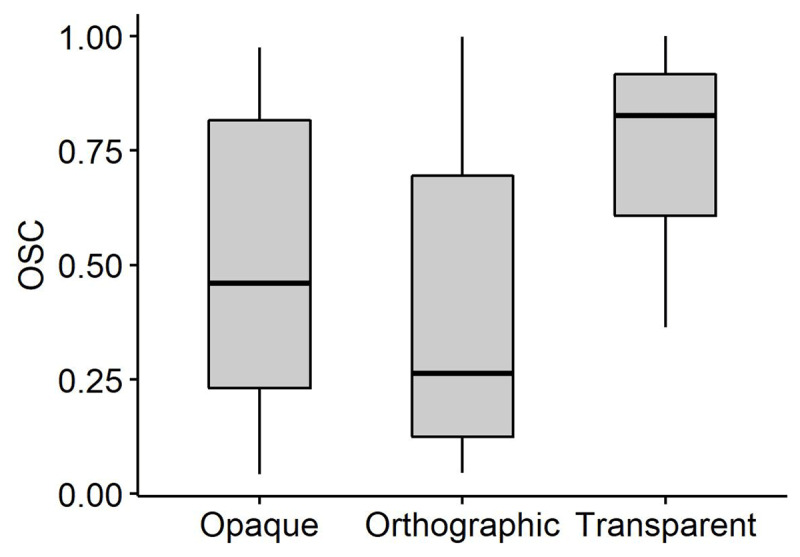
OSC distribution for the transparent, opaque and orthographic English target stems.

As stated in the Introduction, we also wanted to assess the role of Orthography–to–Semantics Consistency (OSC) in L2, and particularly whether this variable affects morphological priming. Because OSC typically co–varies with morphological transparency (Marelli et al., 2015), we first checked whether this was the case also in our set of stimuli, as indeed it was (see Figure 9; *F*[2, 144] = 21.02*, p <* .0001). We thus simply substituted *morphological type* with OSC in the proficiency models that yielded significant priming modulations with the former variable.

**Figure 10:**
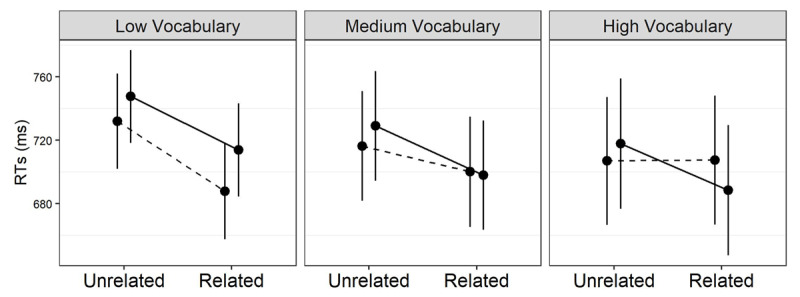
Model–based estimates of response times (RTs) relative to the interaction between prime relatedness, OSC, and vocabulary in L2. Effects are estimated at the 5th, 50th (median) and 95th percentile of the vocabulary distribution, and at the 20th (dashed line) and 80th percentile (solid line) of the OSC distribution. Error bars are 95% confidence intervals.

**Figure 11:**
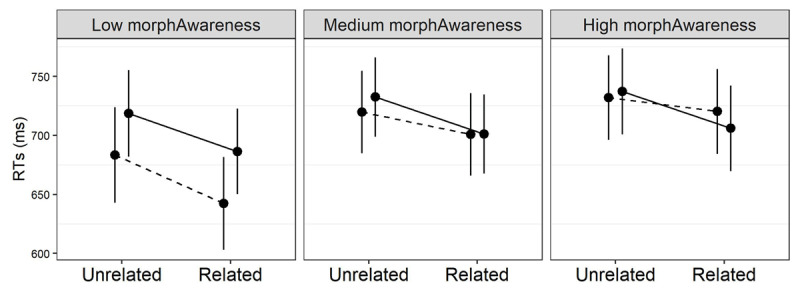
Model–based estimates of response times (RTs) relative to the interaction between prime relatedness, OSC, and morphological awareness in L2. Effects are estimated at the 5th, 50th (median) and 95th percentile of the morphological awareness distribution, and at the 20th (dashed line) and 80th percentile (solid line) of the OSC distribution. Error bars are 95% confidence intervals.

OSC modulates morphological priming in L2, via interactions with vocabulary, *χ^2^*[1] = 181.84*, p <* .0001 and morphological awareness, *χ^2^*[1] = 111.53*, p <* .0001. Phonemic discrimination does not seem to modulate priming significantly in the OSC model, *χ^2^*[1] = .27*, p* = .6. Figures 10 and 11 illustrate these interactions, which both show the same pattern: priming remains strong independent of the proficiency metrics when OSC is high, but shrinks toward zero with growing vocabulary and morphological awareness when OSC is low. Given that high OSC characterizes target words in the transparent condition and low OSC marks target words in the opaque and orthographic conditions (see Figure 9), these results essentially mirror those that emerged with vocabulary and phonemic fluency above – transparent priming (i.e., priming at high OSC) is independent of proficiency, while orthographic and, to some extent, opaque priming (i.e., priming at low OSC) decreases with increasing proficiency.

In terms of how well OSC accounts for the priming pattern as compared to the classic distinction between transparent, opaque, and orthographic primes, we computed the Akaike Information Criterion (AIC; Akaike, 1974) for the category-based and OSC models involving the proficiency metrics that were significant with both approaches, that is, morphological awareness and vocabulary. In both cases, AIC is lower for the OSC models (113613.5 vs. 111639.7, and 113623.8 vs. 111662.4, for morphological awareness and vocabulary, respectively). These results suggest that OSC provides a better account for the data than the categorical distinction between transparent, opaque, and orthographic primes.

## 4. Discussion

In this study we show that orthographic and morphological priming differs in L1 and L2. In L1, we replicated the widely attested pattern whereby the recognition of a target word is facilitated by the prior presentation of a semantically transparent (e.g., dealer-DEAL) or semantically opaque (e.g., corner-CORN) prime, but not by a non–morphological, orthographic prime (e.g., public-PUB). In L2, genuine and opaque derivations provide facilitation, similarly to L1. However, form primes also provide facilitation, contrary to the native language. We also found that transparent derivations yield more priming than opaque primes, both in L1 and L2.

Critically, we discovered that this group-level pattern in L2 is modulated by readers’ proficiency, as tracked by phonemic discrimination, vocabulary, and morphological awareness. While transparent priming remains consistently strong, facilitation in the form condition decreases with increasing phonemic discrimination and vocabulary skills. Opaque priming also shrinks with growing vocabulary, but less than form priming. Because both morphological conditions seem to behave differently from the orthographic baseline, this confirms the genuine morphological nature of this effect. Morphological awareness modulates opaque priming and we find no evidence that any other proficiency metric is specifically related to morphological priming. Age of Acquisition also seems to play little or no role, as attested also in Veríssimo et al. (2018).

Finally, we observed that Orthography–to–Semantic Consistency (OSC) affects lexical decision in a second language, and extend the growing body of evidence for OSC effects in L1 (Marelli et al., 2015; Amenta et al., 2017). Interestingly, we found that OSC also interacts with individual proficiency, and is able to account for morphological priming as well as (or better than) the classic distinction between transparent, opaque, and orthographic primes (Amenta et al. 2020).

At the group level, our results seem to confirm a pattern of facilitation often reported in previous studies – transparent primes (e.g., *dealer-DEAL*) provide more facilitation than orthographic controls (e.g., *dialog-DIAL*), while opaque primes (e.g., *corner-CORN*) stand somewhere in between. Unsurprisingly, the statistical pattern is clearer for L1 than for L2. For L2, proficiency analyses (discussed below) provide crucial insight and therefore the group-level pattern is not particularly important. More interesting is the significant difference between transparent and opaque priming in L1-Italian. This replicates the pattern reported by Marelli et al. (2013) for this language, and confirms that at least some morpho-semantic processing happens early during the visual identification of complex words, in line with other priming results (e.g., Feldman et al., 2012; Jared et al., 2017), a meta-analysis of masked priming data (Davis and Rastle, 2010; Feldman et al., 2009), and evidence from other paradigms (e.g., Amenta et al., 2015; Schmidtke et al., 2017). Amenta et al. (2020) recently showed that masked morphological priming interacts with OSC in L1, which also suggests some early semantic processing triggered by a morpho-orthographic analysis. Moreover, Amenta et al’s results show that dynamics in the lexical-semantic network qualify the broad distinction between transparent and opaque words in important ways. We come back to this point below, when we discuss the OSC analysis reported in this paper.

It is also important to note that we found opaque priming to be stronger than orthographic facilitation in L1-Italian, which again confirms the data by Marelli et al. (2013) and shows that, even if some morpho-semantic processing is likely in place, morphological segmentation happens independently of semantic transparency. This result is in keeping with data from several other languages (e.g., Diependaele et al., 2011; Kazanina, 2011; Longtin et al., 2003; Marslen-Wilson et al., 2008; Rastle et al., 2000), although it must be noted that Milin et al. (2017) recently reported a weak priming effect for pairs with substantial orthographic overlap, independent of the presence of a suffix.

Again at the group level, our results seem to support the idea that morphological processing during visual word identification differs in L1 and L2. This is in line with Clahsen and Felser (2006), Li et al. (2017), Li and Taft (2019) and, for inflectional priming, Feldman et al. (2010). This finding also seems to contradict Diependaele et al. (2011). However, if one looks closely at the priming pattern observed in the individual morphological conditions, the inconsistency is less than it would seem. Genuine derivations provide solid facilitation in both L1 and L2, which is consistent across studies. In our experiment, opaque primes tend to yield less facilitation than transparent derivations in L2 (as they also do in L1) – again, this is comparable to the observations in Diependaele et al. (2011). L1 and L2 differ most in form priming, with a clear null effect in L1, in line with a large body of literature (e.g., Rastle et al., 2004; Longtin et al., 2003; Forster et al., 1987), and clear orthographic similarity facilitation in L2. Again, this pattern of results mirrors the findings of Diependaele et al. (2011).

The main difference between Diependaele et al. (2011) and our observations here is in the comparison between form and opaque priming in L2 – opaque priming is somewhat smaller in our experiment (19 ms vs 26 ms), which makes it harder to differentiate from form priming, while these two conditions were statistically distinguishable in Diependaele et al. (2011) (albeit only when the two groups of L2 learners that they considered were analysed together, showing that this effect was not very strong even in their data). Overall, then, despite a different outcome in the three–way interaction between *prime relatedness, morphological type* and *language*, the difference between our data and Diependaele et al. (2011) only consists in a somewhat weaker L2 opaque priming in the current experiment.

L2 form priming is clearly solid here, as it was in previous experiments: orthographically similar words facilitate each other during lexical identification in a second language. In line with previous reports, this contrasts with a very clear null effect in L1. This pattern nicely mirrors the Prime Lexicality Effect (PLE) observed in native speakers (e.g., Forster and Veres, 1998) – nonwords provide strong facilitation to orthographically related targets in masked priming (e.g. *contrapt*–*CONTRAST*), but this facilitation is reduced, and sometimes even turns into inhibition (Davis and Lupker, 2006), when the prime is a real word (e.g., *contract*–*CONTRAST*). This phenomenon is classically interpreted in terms of lexical competition – both *contrapt* and *contract* would provide the same amount of facilitation at the letter coding level, but the established lexical representation for *contract* would then compete with that of the target word, thus generating lexical inhibition that would offset the sub–lexical priming.

From this perspective, our data suggest that L2 words behave similarly to nonwords in L1, mimicking the results obtained in native speakers by Grainger and Beyersmann (2020). L2 lexical representations might not be very well established (or even present). Therefore, lexical competition would be reduced (or absent), thus providing no offset to the sub–lexical facilitation brought about by a form–related prime. This nicely connects with the growing literature on novel word learning (e.g., Gaskell and Dumay, 2003; Sobczak and Gaskell, 2019; Tamminen and Gaskell, 2008; Tamminen et al., 2010; Walker et al., 2019), where lexical competition is often taken as the primary diagnostic for a fully consolidated lexical memory.

This hypothesis also fits neatly with the proficiency analysis, particularly concerning phonemic discrimination and vocabulary: L2 form priming shrinks with growing competence on these two skills. This may suggest that L2 lexical memories become more fully established with growing proficiency, such that lexical competition is progressively more obvious as readers gain command over a second language.

More generally, this interpretation is also consistent with recent explanations of nonword morphological priming in L1. Grainger and Beyersmann (2017) suggest that the reason why non-suffixed nonwords (e.g., *farmald*) prime their stems (*FARM*) while analogous word primes fail to do so (e.g., *dialog-DIAL*) is exactly that the former lack lexical representations, and therefore provide no inhibition to their targets. Here we connect this interpretation to the construct of lexicalization/consolidation of novel word memories, therefore providing a general framework to account for both nonword processing and word learning in L1, and lexical processing along a proficiency continuum in L2. Grainger and Beyersmann (2017) also center their model around activation of embedded word units. This mechanism provides an alternative account for L2 orthographic priming; in addition to form similarity due to shared letters, *freeze* might facilitate the processing of *free* because the visual word identification system has recognized the target as an embedded word within the prime. Note, however, that this alternative interpretation does not affect the core idea that words fail to provide strong inhibition in L2: If form priming is to surface, *freeze* must not compete strongly with *free*, no matter where the facilitatory side of the effect comes from.

Before moving to the individual proficiency effects, a word of caution is in order for the group-level comparison between L1 and L2 priming. Our within-participant design did not allow for an L1-L2 comparison within the same language. Of course, this leaves the possibility open that the difference we observed here is related to the specific features of Italian and English, rather than to their L1/L2 status, or to the different sets of items that we used. Although we took great care in making the L1-Italian and the L2-English stimulus sets as similar as possible, it will be important to see our findings replicated with a different design, or with other languages.

The impact of individual readers’ proficiency – as tracked by phonemic discrimination, vocabulary, and morphological awareness – extends well beyond the orthographic condition, and critically qualifies the entire pattern of form and morphological priming.

Consistently across the three different metrics, the effect of transparent primes appears to be rather insensitive to proficiency. Also consistent across metrics, visual word identification is dominated by mere form similarity when readers do not have great command over their L2. As the leftmost panels of Figures 4, 5 and 6 clearly show, transparent, opaque and orthographic primes have barely-distinguishable effects on their (pseudo–)stems at low levels of proficiency. This pattern resembles the results reported for the inflectional domain by Feldman et al. (2010), where lower proficiency readers failed to show any different facilitation for morphological and orthographic primes.^7^ As with our suggestion above, this points to a rather weak lexical network in low–proficiency L2 readers. At this stage, the lexicon may perhaps be characterized more as a collection of unconsolidated word memories than as a network that supports the lexical dynamics typical of L1.

These results suggest that morphological priming is a convenient metric to track the emergence of a fully–fledged (i.e., L1–like) morpho–lexical system in a second language. Early on, form similarity would be the only driving force, with no morpho–lexical distinction between orthographic, opaque, and transparent priming. As word representations become more and more consolidated, lexical competition arises, driving down purely orthographic priming. Genuine morphological detectors would also start to develop, so that complex words progressively differentiate from an orthographic baseline, eventually yielding a pattern of facilitation that closely resembles L1.

Where do opaque primes stand in this framework? Interestingly, different proficiency metrics seem to provide somewhat different answers here. For example, opaque priming fits neatly with transparent priming in the interaction with phonemic awareness. This resonates with group-level accounts of morphological priming that show no difference at all between genuine and pseudo-derivations (e.g., Kazanina, 2011; Longtin et al., 2003; Marslen-Wilson et al., 2008; Rastle et al., 2004). On the contrary, the interaction between masked priming and phonemic discrimination shows that, while opaque priming is still significantly different from the orthographic baseline (thus confirming the genuine morphological nature of these effects), it also differs from the transparent condition: the facilitation from *corner* to *corn* shrinks more with growing proficiency than the facilitation from *dealer* to *deal*. This pattern resonates with accounts of group-level effects in early morphological processing that grant some role to semantics (e.g., Feldman et al., 2009; 2012; Jared et al., 2017).

The pattern revealed by morphological awareness is also intriguing: the higher the score on this metric, the smaller the opaque facilitation (whereas form and transparent priming are largely unaffected). Thus, it seems that this skill helps the reader distinguish between genuine derivations and words that only have an orthographic appearance of complexity. Since morphological awareness taps into one’s capacity to manipulate word parts in a meaningful way (and therefore is strongly based on semantics), this connection may not be surprising. However, morphological awareness is also related to production more than to comprehension, and requires explicit judgments, so that participants have to deliberately access their morphological knowledge. This stands in stark contrast with masked priming, which taps into early perception and implicit/unaware processing. Nevertheless, the data illustrated here suggest some connection between the two tasks that surely calls for more investigation.

The critical importance of each reader’s proficiency profile in these data also relates to: (i) the mounting evidence on the effect of individual variability in L1 (e.g., Andrews and Hersch, 2010; Burt and Tate, 2002; Andrews and Lo, 2013; Beyersmann et al., 2015a); (ii) proficiency studies in developing readers which show different morphological priming profiles according to vocabulary and spelling skills (Beyersmann et al., 2015b); and (iii) developmental data that point to some changes over the course of adolescence in the way letter strings are processed (Dawson et al., 2017). Evidence is growing that experience with the written language (and, possibly as a consequence, better and more refined orthographic representations/processing) produces substantial change in the dynamics behind visual word identification. A precise characterization of the cognitive profile of each individual reader and a careful consideration of their experience with visual words is increasingly fundamental to the field, because it seems to critically qualify most of the phenomena previously believed to emerge in undistinguished groups of participants. Particularly relevant here is the recent suggestion that readers with relatively lower lexical and orthographic representations/processing may rely more on morphological structure (e.g., Beyersmann et al., 2015a; Grainger and Beyersmann, 2017). This is very consistent with the general idea, fully supported by the present data, that morphological processing is modulated by language proficiency. Beyersmann’s and Grainger’s work focused on L1; here we extend the idea to L2.

Other recent evidence shows that developing readers rely more on morphological processing when their language is less consistent in its spelling-to-sound relationships (Mousikou et al., 2020; Beyersmann et al., 2020). Although these data focus more on the sub-lexical stages of reading and visual word identification, they reinforce the idea that morphological processing is modulated by the availability and/or quality of other levels of representation that readers might use.

Among the many proficiency indices we considered here, vocabulary, phonemic discrimination and morphological awareness turned out to be the best metrics to account for L2 morphological priming. We would not want to draw bold conclusions based on these data. There was not much evidence in the L2 literature on individual proficiency scores and morphological priming, and therefore it was difficult to make specific predictions; our approach was largely an exploratory one. Furthermore, the proficiency metrics were somewhat correlated with each other. This is probably unavoidable given the complexity underlying one’s knowledge of a language (e.g., Leclercq et al., 2014). We took great care to ensure that this set of predictors was indeed mapping independent constructs (see the results of the PCA illustrated above, in Figure 3). However, we cannot entirely rule out the possibility that at least part of the priming modulation that we observed here emerged spuriously as a consequence of this dense network of correlations. A few considerations would suggest otherwise, though. For example, the three significant predictors do not seem to correlate particularly strongly as compared to other proficiency metrics; the correlation coefficients between phonemic discrimination and morphological awareness (.43) and between phonemic discrimination and vocabulary (.44) sit around the lower quartile of the distribution. Also, morphological awareness is most strongly tight to oral comprehension and spelling, neither of which modulates priming.

An obvious comparison for these data is the L1 results obtained on the issue by Andrews and Lo (2013), Andrews and Hersch (2010) and Beyersmann et al. (2015a). These findings focus particularly on vocabulary and spelling skills, and on their relative strength. Using a more bottom-up, exploratory approach, our analyses highlight that vocabulary modulates priming in L2, in line with some L1 reports (Beyersmann et al., 2015a), although not all (Andrews and Lo, 2013). We found no evidence for a specific role of spelling in the modulation of masked morphological priming in L2; again, this is in line with Beyersmann et al. (2015a), but not with Andrews and Lo (2013). Generally speaking, it is perhaps not surprising that L1 and L2 results do not entirely match. There is evidence, in fact, that native and non-native language processing recruit at least partly different neural systems and cognitive mechanisms (Sulpizio et al., 2020; Liu and Cao, 2016). Therefore, any comparison should be interpreted with some caution. However, we note that, in this specific case, there seems to be more inconsistency in the L1 data themselves than in the comparison between L1 and L2.

Why should these particular proficiency metrics affect morphological and form priming specifically? While the role of morphological awareness in the modulation of opaque priming is fairly obvious (see above), it is less easy to understand why vocabulary and phonemic discrimination should be big players here. We can only speculate at this stage, but for what concerns vocabulary, one possibility relates to the proposal by Beyersmann et al. (2015a) and Grainger and Beyersmann (2017); readers with a weak and relatively small lexical network might rely more heavily on other sources of information, such as morphology. This account, however, would be hard to extend to phonemic discrimination, which should not be particularly important in the quick and automatic identification of visual words entailed by masked priming. More research is clearly needed here, to complement our exploratory approach with some more specific confirmatory experiments.

Finally, we demonstrated for the first time an effect of Orthography–to–Semantic Consistency (OSC; Marelli et al., 2015) in L2, showing that readers capture fine-grain, probabilistic ties in form–to–meaning mapping outside of their native language. This result invites an intriguing new perspective on the learning of a second language, which may be related (among other things, of course) to an appreciation of the statistical structure behind the relationship between form and meaning in the novel lexicon (Forster and Veres, 1998; Castles et al., 2007; Perfetti and Hart, 2002; Perfetti, 2007; Andrews and Hersch, 2010; Andrews and Lo, 2013; Hersch and Andrews, 2012). Further reinforcing this suggestion, we found that sensitivity to OSC interacts with proficiency – the more one gains command over L2, the more sensitive it becomes to probabilistic relationships between orthography and semantics.

This, in turns, affects the priming pattern. When proficiency is low, priming is strong across the board, that is, independent of OSC. When proficiency is high, instead, priming is critically qualified by OSC, so that facilitation disappears for targets with lower values on this metric. One possible account for this result is that, when the target word comes from a lexical region where the correspondence between form and meaning is largely arbitrary (i.e., OSC is low), participants discount form as a source of information to meaning. Thus, they are left with purely lexical-orthographic processing, where facilitation coming from the shared letters is offset by the lexical competition between the prime and target representations. When OSC is high, the participants’ visual identification system knows that form does indeed point to meaning, and therefore the (consistent) semantic information coming from the prime provides a headstart in processing the target. This sensitivity to the structural characteristics of the lexical space, however, only emerges when L2 proficiency is high.

The data described here also suggest that OSC provides a nice account of the priming pattern independent of the classic categorical distinction between transparent, opaque, and orthographic primes (see Amenta et al., 2020, for similar evidence in L1). OSC correlates with these categories, and it proved able to account for priming in the statistical model even when these other predictors were removed. The AIC analysis also suggests that OSC provides a better fit for the data than the classic categorical approach. This may necessitate a different interpretation of morphological priming, which would depend not only (or at all?) on the relationship between primes and targets themselves, but on transparency in form–to–meaning mapping in the lexical region from whence the target and the prime come (Amenta et al., 2020). This item-level, rather than category-level account of priming resonates with Milin et al. (2017), who found that, overall, most variability in priming was accounted for by between-prime variation, rather than a systematic distinction between classes of prime-target relationships.

These considerations are relevant for the debate around the role of semantics in the early stages of visual word identification (e.g., Amenta and Crepaldi, 2012; Davis and Rastle, 2010; Feldman et al., 2012). First of all, it might justify the different results that have sometimes been reported across experiments, particularly in different languages where form–to–meaning mapping might easily be more or less consistent (e.g., Kazanina, 2011; Longtin et al., 2003; Feldman et al., 2012). Most importantly, an effect of OSC in masked priming unequivocally requires *both* early access to semantic information, as form–and–meaning accounts would suggest (e.g., Feldman et al., 2012; Milin et al., 2017), *and* the activation of all the input’s possible orthographic units, independent of whether these units will turn out to be meaningful in any given word, which is the fundamental tenet of form–then–meaning theories (Rastle et al., 2004; Crepaldi et al., 2010; Taft and Nguyen-Hoan, 2010). The focus of the theoretical debate would move beyond the issue of whether semantics play an early role in complex word identification; the answer to this question would be double-edged, as meaning does play a role, in the sense that semantic information is accessed, but at the same time it doesn’t, because orthographic units are identified and activated independently of their transparency (e.g., Amenta et al., 2015). A more important issue would be *how* form and meaning play their double act; OSC provides an initial cue, but there are many questions that will need to be addressed, such as determining the relevant orthographic neighbourhood that defines consistency (e.g., Marelli and Amenta, 2018), and evaluating how much this is based on morphology itself versus being a more general mechanism that identifies any possible form–meaning regularity. As Amenta et al. (2020) suggest, the data currently available might not yet be sufficient to justify a comprehensive reorientation towards the OSC approach; after all, this variable correlates strongly with the classic categorical distinction between transparent, opaque, and orthographic items, and it remains difficult to tease the two sides apart. Nevertheless, the present data, and the growing body of OSC effects that is accumulating (e.g., Amenta et al., 2017; Marelli and Amenta, 2018), surely points in this direction.

More generally, OSC highlights the role of the structural characteristics of the lexical-semantic space whence words come, and particularly the probabilistic, associative cues that tie representations together in this space. This marries well with some recent data in L1. For example, Grainger and Beyersmann (2020) showed that morphological priming is modulated by the conditional affix probability of the embedded word, i.e., how likely it is in the language to find an affix after a given stem/embedded word. These data demonstrate that priming is sensitive to lexical/morphological regularities, which, as the present L2 data suggest, might be acquired through experience with a given language, either native or non-native. From this perspective, priming would seem to depend on the generation of predictions in visual word recognition, guided by prior linguistic input and, again, the appreciation of probabilistic associative cues.

## Data accessibility statement

All data, stimuli and code that were used in the context of this experiment are openly available at the Open Science Framework.

## Additional File

All the materials related to this paper (the stimulus list, the data sets, the analysis scripts, and the tools that we used during the analysis) are publicly available at the Open Science Framework (https://osf.io/jnrvy/).

10.5334/joc.221.s1 Appendix. L1 – Italian and L2 – English.

## Footnotes

In this manuscript, we refer to words such as brothel-BROTH or corner-CORN as having a “morphologically opaque” relationship. This term puts the emphasis on the absence of semantic link between prime and target, however we acknowledge that researchers also use the term “pseudo-morphological” due to the prime having a portion of the word which resembles a morpheme, but is not (i.e., in the word *corner*, the segment *-er* it’s a pseudomorpheme).

Foote (2015) also included proficiency in her experimental design. However, this study investigates speech production in the context of a phrase completion task, which taps into cognitive processes that are very different from those driving masked visual priming. Therefore, we do not describe these data in more detail.

We checked that the pattern of results did not change when the exactly the same participants were included in the L1 and L2 datasets (see below in the Results section).

We also examined the Inverse Gaussian distribution, which had an equivalent quality of fit. However, we eventually opted for the Gamma distribution because models converged more easily.

To check that the pattern of results did not change when exactly the same set of participants were considered in the L1 and L2 experiments, we refitted the models above excluding from the Italian dataset those participants who were excluded from the English dataset. Indeed, the pattern of results remains the same.

*Phonemic fluency* and *reading comprehension* were fairly close to the significance threshold (*p* =*.16* and *p* = *.09*, respectively). We therefore explored these effects slightly more in depth in the analysis script, which the interested reader can find at the Open Science Framework repository for this project. While we do not consider these effects as statistically reliable and therefore do not discuss them further, the reader may explore the data and perhaps find interesting information for future research.

Interestingly, it is not entirely clear whether morphological and orthographic primes were different from an unrelated baseline in Feldman et al. (2010), while these primes clearly yield quicker responses than unrelated controls in the present work.
