# Supplementary material for: Masked Morphological Priming and Sensitivity to the Statistical Structure of Form–to–Meaning Mapping in L2
Source: J Cogn. 2022 May 9;5(1):30. doi: 10.5334/joc.221 (PMC9400631; doi:10.5334/joc.221)
Supplement: Appendix. — L1 – Italian and L2 – English. [file joc-5-1-221-s1.pdf]

Appendix

L1 – Italian

	**Transparent condition**	
Target	Related prime	Control prime
ARCO	arcata	melone
ARTE	artista	sottile
ASMA	asmatico	fogliame
ASTRO	astrologo	signorile
ATTO	attore	morale
BANCA	bancario	minerale
BENDA	bendaggio	minerario
CALCIO	calciatore	ventricolo
CAMPANA	campanile	variabile
CANTO	cantore	mammola
CREMA	cremoso	fertile
CUBO	cubista	tessile
DELFINO	delfinario	carotaggio
DITO	ditata	idrico
DOSE	dosaggio	camerata
ERBA	erboso	areola
FAMA	famoso	ideale
FANGO	fangoso	porcile
FARINA	farinoso	adrenale
FATO	fatale	fidata
FIENO	fienile	vettore
FORNO	fornaio	frenata
FRUSTA	frustata	ciarpame
GETTONE	gettonato	scambista
GHIACCIO	ghiacciolo	campagnolo
LEGNO	legname	puerile
MAZZA	mazzata	bombola
MITO	mitico	botola
NERVO	nervoso	turista
NOIA	noioso	sadico
OCCHIO	occhiata	naturale
ORIGINE	originario	linguaggio
ORTO	ortaggio	litorale
PAROLA	paroliere	necrotico
PENSIONE	pensionato	petroliera
POLLO	pollame	nudista
REGIA	regista	pittore
SABBIA	sabbiatura	soporifero
SANO	sanitario	alcolista
SASSO	sassata	fazione

SCHIFO	schifoso	monetario
SERVO	servile	sudista
STILE	stilista	liberale
STRADA	stradale	eleganza
TASTO	tastiera	frittata
TAVOLO	tavolata	plateale
TAXI	taxista	pontile
UGGIA	uggioso	spumame
VELLO	veliero	bravata
VETRO	vetrata	fondale
	**Opaque condition**	
Target	Related prime	Control prime
ABITO	abitudine	documento
ARTIGLIO	artigliere	locandiere
BALLO	ballatoio	sedimento
BILE	bilico	barile
BRIGA	brigante	revisore
CALVO	calvario	lebbroso
CARRO	carriera	fiorente
CAVIA	caviale	lunario
CERNIA	cerniera	sciabola
COLLE	collezione	parcheggio
CONO	conato	senile
COSCIA	coscienza	comunista
COSTA	costanza	pigmento
COSTO	costume	normale
DOGA	dogana	urbana
FALCO	falcata	corroso
FIRMA	firmamento	bilanciere
FORMA	formaggio	simpatico
FORZA	forziere	pompiere
FOSSO	fossile	calcolo
GARA	garante	padrone
GELO	geloso	dorato
GENERO	generoso	pazienza
GESTA	gestazione	sventurato
GOMITO	gomitolo	capienza
GRANO	granito	radioso
INDOLE	indolenza	discepolo
MAESTRA	maestranze	vivandiere
MASSO	massaggio	artistico
MATTO	mattanza	plenario
MIMO	mimosa	tisana
ORMA	ormeggio	timoroso
OSTE	ostaggio	acquario

PIETA'	pietanza	stellata
PIGNA	pignolo	festivo
QUIETE	quietanza	vituperio
RETE	retaggio	lampante
RETTA	rettile	violino
SALE	salario	formale
SERENA	serenata	volubile
SOSTA	sostanza	alleanza
STIVA	stivale	europeo
TATTO	tattico	caldaia
TEMPERA	temperanze	plafoniera
TESTA	testamento	cioccolato
TRATTO	trattore	scuderia
VANTO	vantaggio	piacevole
VENTO	ventola	pelvico
VINO	vinile	embolo
VIOLA	violenza	opinione

	**Orthographic condition**	
Target	Related prime	Control prime
ALBERO	albergo	istinto
AVO	avorio	patria
BANDA	bandiera	convento
BARRA	barracuda	cespuglio
BOCCA	boccia	sobria
CAMBIO	cambusa	ridosso
CAVO	cavallo	codardo
CELLA	cellula	relitto
CLAVA	clavicola	prematuro
CONGRUO	congrega	obsoleta
CORDA	cordoglio	travaglio
CORO	corallo	baruffa
CORTE	corteccia	scongiuro
FARO	faringe	omicida
GUADO	guadagno	ridicola
GUANO	guanto	stalla
LAMA	lamento	monello
LANA	lancia	radice
LENZA	lenzuola	cardiaco
LUCE	lucertola	dinosauro
LUPO	lupara	frolla
MALE	malta	riffa
MANDRIA	mandrillo	demoniaco
MANO	manto	spola
MASSA	massacro	collasso

MERCE	mercurio	castagno
META	metallo	dipinto
MUSEO	museruola	idilliaco
OBLIO	obliquo	cruento
ORDINE	ordigno	ristoro
PALLA	pallido	storico
PASSERO	passerella	salmonella
PELLE	pellicola	pagamento
PIANO	pianeta	salotto
PRODE	prodigio	prefisso
RAGGIO	raggiro	colosso
RESTO	restauro	vergogna
RISO	riserbo	ghianda
SALA	salasso	frangia
SALAME	salamandra	malaugurio
SCALO	scalogno	sonaglio
SCAMPO	scampolo	ossequio
SCIA	sciame	staffa
SOFFIO	soffitto	clausola
SPIA	spiaggia	orologio
SPINA	spinaci	litigio
SQUALO	squallido	trapianto
TRAMA	tramonto	sostegno
TRIBU’	tribuna	lattice
VELA	velcro	olezzo

L2 – English

	**Transparent condition**	
Target	Related prime	Control prime
ACID	acidic	yearly
ACRE	acreage	plunder
ADOPT	adopted	kingdom
AGREE	agreement	equipment
ALARM	alarming	composer
ANGEL	angelic	watcher
ARTIST	artistry	calmness
BARON	baronet	voucher
BEARD	bearded	thinker
BLOOD	bloody	active
BOMB	bomber	lessen
BULB	bulbous	leftist
CHILL	chilly	finely
CLOUD	cloudless	enactment
CREAM	creamy	watery

CRITIC	critical	tendency
DIET	dietary	wearily
DREAM	dreamer	masonry
DRUNK	drunkard	feathery
EMPLOY	employer	addition
ERUPT	eruption	vicarage
FILTH	filthy	harden
FIZZ	fizzle	touchy
FLESH	fleshy	lovers
FLOAT	floater	missive
GLOOM	gloomy	miller
GOLF	golfer	thinly
GOVERN	government	situation
GREEN	greenery	snobbish
GUILT	guilty	formal
INHIBIT	inhibitory	amateurish
LEGEND	legendary	anxiously
MARSH	marshy	thorny
MOURN	mourner	tripper
NORTH	northern	friendly
NYMPH	nymphet	acutely
OXYGEN	oxygenate	fossilise
POET	poetry	dealer
QUIET	quieten	mimicry
REACT	reaction	physical
RENEW	renewable	exemption
RISK	risky	downs
SCALD	scalding	jauntily
SOFT	soften	heroic
TEACH	teacher	finally
TOAST	toaster	wishful
TRAIN	trainee	cookery
TUFT	tufted	silken
VIEW	viewer	ranger
WIDOW	widowed	beastly
	**Opaque condition**	
Target	Related prime	Control prime
AMEN	amenable	palpably
AMP	ample	widen
ARCH	archer	feudal
AUDIT	audition	selfless
BOARD	boarder	factual
BRAND	brandy	safely
BRISK	brisket	foundry
BUZZ	buzzard	loyally

COAST	coaster	muffler
COUNT	country	service
COURT	courteous	developer
CRAFT	crafty	vainly
CROOK	crooked	pottery
CRYPT	cryptic	dweller
DEPART	department	production
DISC	discern	starter
EARL	early	within
FACET	facetious	distantly
FLEET	fleeting	simplify
FLICK	flicker	adviser
FRUIT	fruitless	alcoholic
GLOSS	glossary	sufferer
GLUT	gluten	bridal
GRUEL	grueling	existent
HEART	hearty	folder
HELM	helmet	brutal
INFANT	infantry	validity
INVENT	inventory	murderous
IRON	irony	sandy
LIQUID	liquidate	extremism
NUMB	number	really
ORGAN	organic	leaflet
PLAN	planet	editor
PLUCK	plucky	winger
PLUM	plumage	broiler
PUTT	putty	fishy
QUEST	question	actually
RATION	rational	steadily
SCULL	scullery	narrowly
SECRET	secretary	obviously
SIGN	signet	frosty
SNIP	sniper	hourly
SPLINT	splinter	idealism
STILT	stilted	gaseous
THICK	thicket	scruffy
TREAT	treaty	angler
TROLL	trolley	naughty
TRUMP	trumpet	chatter
UNIT	united	others
WHISK	whisker	coyness
	**Orthographic condition**	
Target	Related prime	Control prime
AGAIN	against	perhaps

APPEND	appendix	believer
ARSE	arsenal	timidly
BASIL	basilisk	benignly
BROTH	brothel	warfare
BUTT	button	prayer
CANDID	candidacy	epileptic
COLON	colonel	ability
COMMA	command	equally
DEMON	demonstrate	instruction
DIAL	dialog	lately
ELECT	electron	suburban
ETHER	ethereal	rumbling
EXTRA	extract	justify
FORCE	forceps	prudish
FREE	freeze	golden
FUSE	fuselage	citation
GALA	galaxy	keeper
GLAD	glade	cuffs
HEAVE	heaven	firmly
INTERN	internation	revolutionary
INVEST	investigate	anaesthetic
JERK	jerkin	twisty
NEIGH	neighbour	struggled
PARENT	parenthesis	lectureship
PHONE	phonetic	dreadful
PLAIN	plaintiff	absurdity
PLUS	plush	filmy
PUB	public	gently
PULP	pulpit	gifted
QUART	quartz	roller
RABBI	rabbit	weekly
SCRAP	scrape	ninety
SHOVE	shovel	tricky
SHUN	shunt	itchy
SIGH	sight	happy
SMUG	smuggle	twelfth
SQUAW	squawk	oddity
STAMP	stampede	defector
STIR	stirrup	buoyant
STUB	stubborn	moisture
STUN	stunt	misty
SURF	surface	medical
SURGE	surgeon	novelty
TACT	tactile	spindly
TEXT	textile	booklet
TWIN	twinkle	cheaply
TWIT	twitch	lesser

VILLA	villain	grossly
WEIR	weird	manly
